# Supplementary material for: A nanopore-based amplicon-sequencing panel to determine the origin and zoonotic host species of Plasmodium knowlesi infections
Source: medRxiv. 2026 Oct 19:2026.10.16.26365752. Preprint. [Version 1] doi: 10.64898/2026.10.16.26365752 (PMC13738823; doi:10.64898/2026.10.16.26365752)
Supplement: Supplementary file 1 [file NIHPP2026.10.16.26365752V1-supplement-1.pdf]

medRxiv preprint doi: https://doi.org/10.64898/2026.10.16.26365752; this version posted October 19, 2026. The copyright holder for this preprint (which was not certified by peer review) is the author/funder, who has granted medRxiv a license to display the preprint in perpetuity.
It is made available under a CC-BY 4.0 International license .

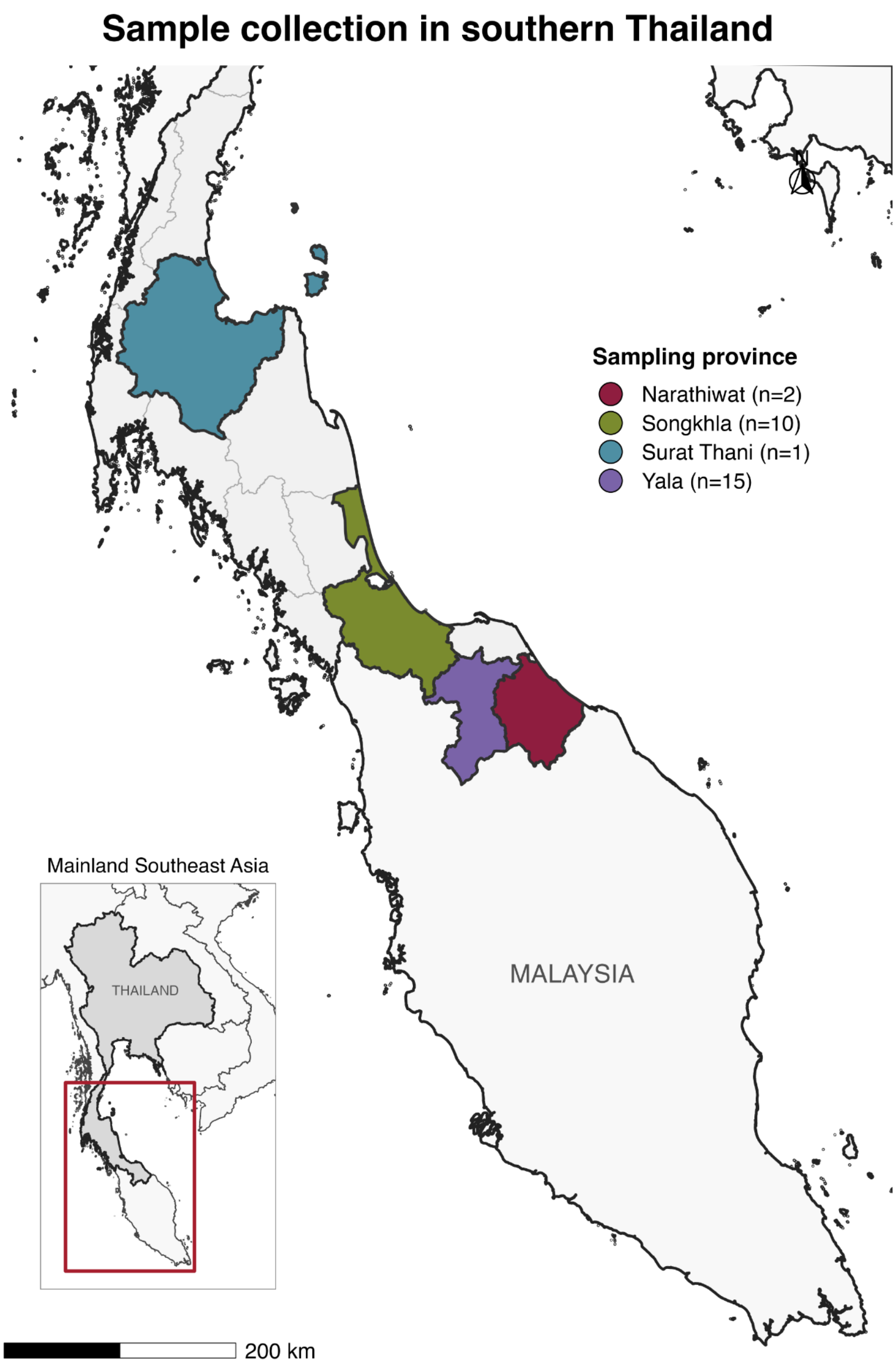

457

458 **Supplementary Figure S1.** Clinical infections sampled in southern Thailand. Map showing the four
459 provinces represented among all 28 sequenced clinical infections: Narathiwat (n = 2), Songkhla (n = 10),
460 Surat Thani (n = 1), and Yala (n = 15). The inset places the study area within mainland Southeast Asia.
461 Province counts refer to the full sequenced infection set; the 23 infections retained for population-
462 genetic analyses are detailed in Supplementary Table 1.

medRxiv preprint doi: https://doi.org/10.64898/2026.10.16.26365752; this version posted October 19, 2026. The copyright holder for this preprint (which was not certified by peer review) is the author/funder, who has granted medRxiv a license to display the preprint in perpetuity.
It is made available under a CC-BY 4.0 International license .

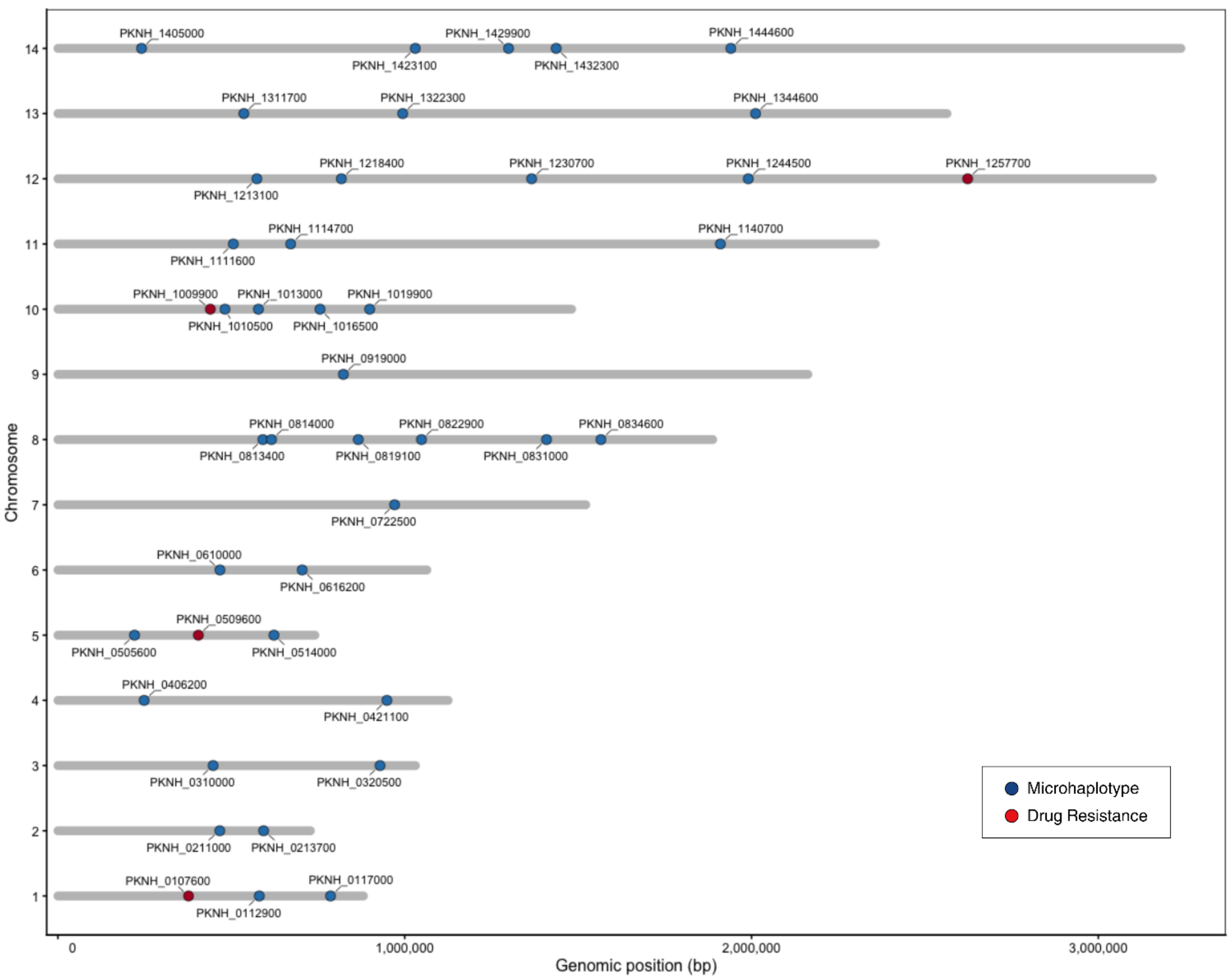

Supplementary Figure S2. Chromosomal distribution and genomic coordinates of the 43 initial candidate amplicons screened during panel development. The candidate set included microhaplotype regions selected from the Peninsular Malaysian WGS diversity screen together with putative antimalarial resistance-gene orthologues and spanned all 14 *P. knowlesi* chromosomes. Thirty primer pairs passed singleplex screening and formed the final experimental multiplex panel; because the only chromosome 9 candidate (PKNH_0919000) failed screening, the final 30-locus panel represents 13 of 14 chromosomes. The Peninsular Malaysian WGS diversity screen used for microhaplotype target discovery was derived from Hocking et al. (2020) (Supplementary Table 2).

medRxiv preprint doi: https://doi.org/10.64898/2026.10.16.26365752; this version posted October 19, 2026. The copyright holder for this preprint (which was not certified by peer review) is the author/funder, who has granted medRxiv a license to display the preprint in perpetuity.
It is made available under a CC-BY 4.0 International license .

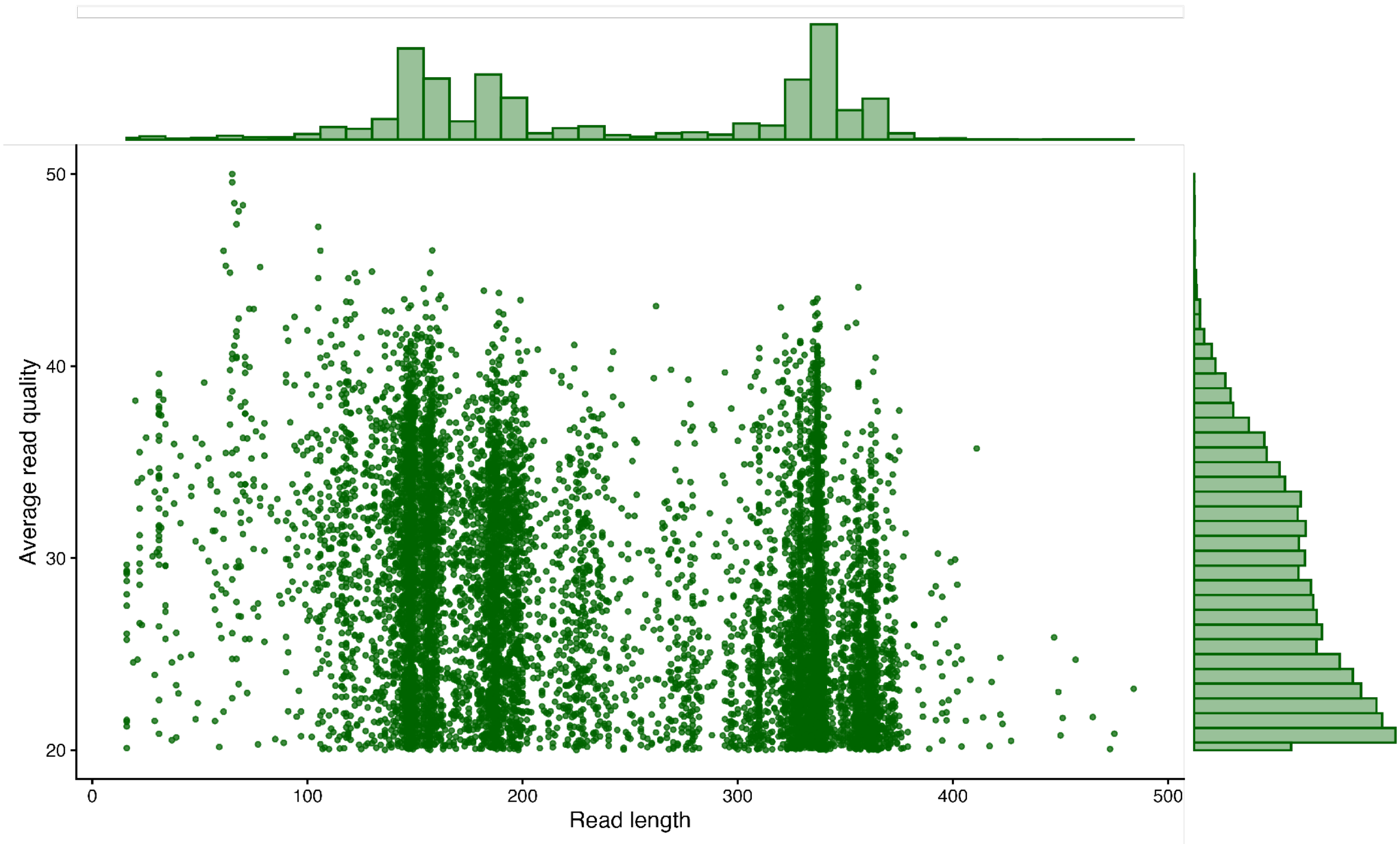

Supplementary Figure S3. Read length and basecall quality distribution for the nanopore AmpSeq run. Scatter plot showing read length versus mean basecall quality, with marginal histograms summarizing the distribution of read lengths and quality scores. The plot was reconstructed from the embedded Plotly data in the NanoPlot HTML report.

medRxiv preprint doi: https://doi.org/10.64898/2026.10.16.26365752; this version posted October 19, 2026. The copyright holder for this preprint (which was not certified by peer review) is the author/funder, who has granted medRxiv a license to display the preprint in perpetuity.
It is made available under a CC-BY 4.0 International license .

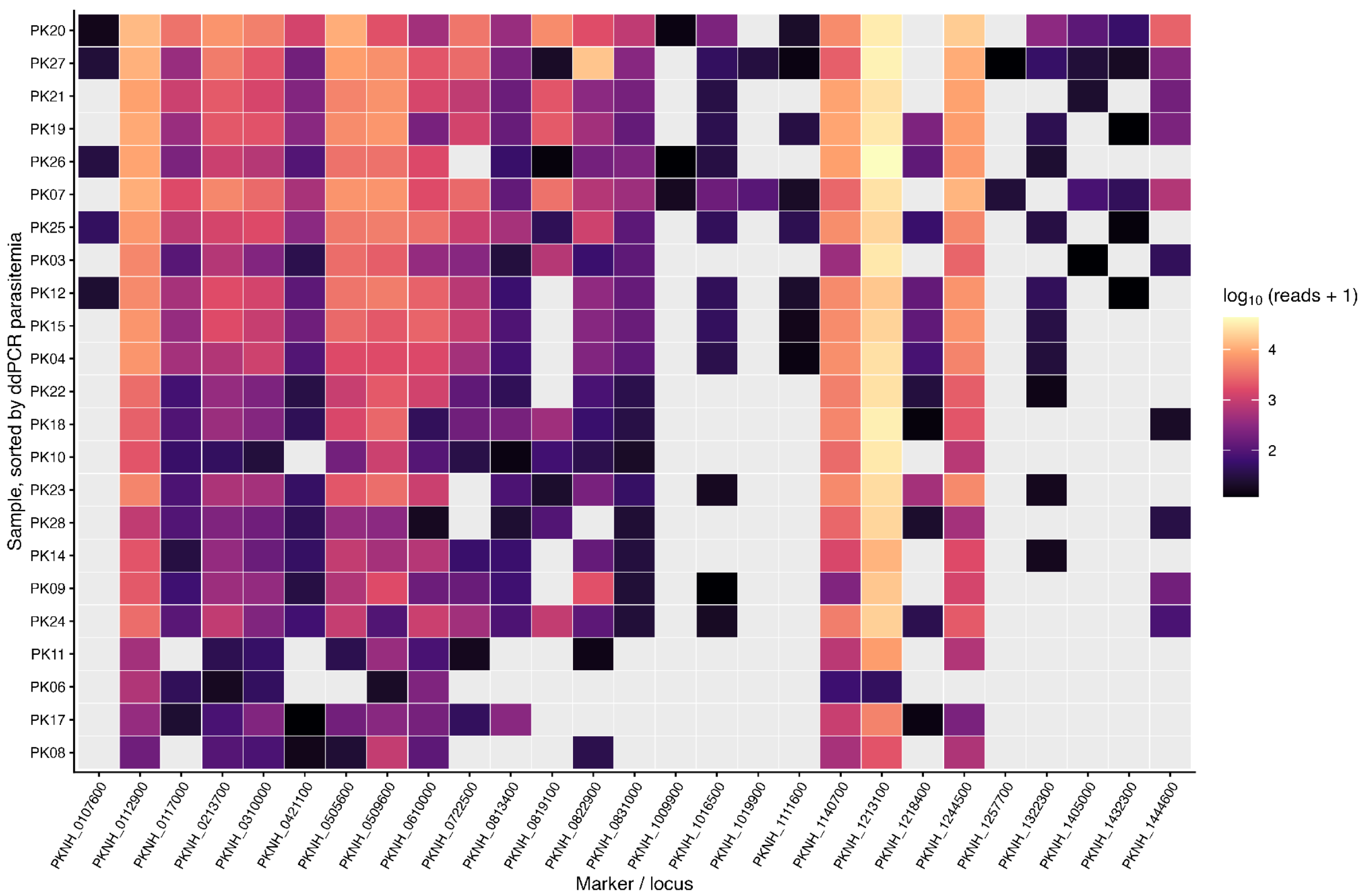

Supplementary Figure S4. Infection-by-locus read recovery across sequenced *P. knowlesi* infections. Heatmap showing summed haplotype-supporting reads per infection and marker from the HaplotypR output. Read depth was calculated by summing reads across retained haplotypes for each infection-marker combination. Grey cells indicate markers with no retained haplotype call.

medRxiv preprint doi: https://doi.org/10.64898/2026.10.16.26365752; this version posted October 19, 2026. The copyright holder for this preprint (which was not certified by peer review) is the author/funder, who has granted medRxiv a license to display the preprint in perpetuity.
It is made available under a CC-BY 4.0 International license .

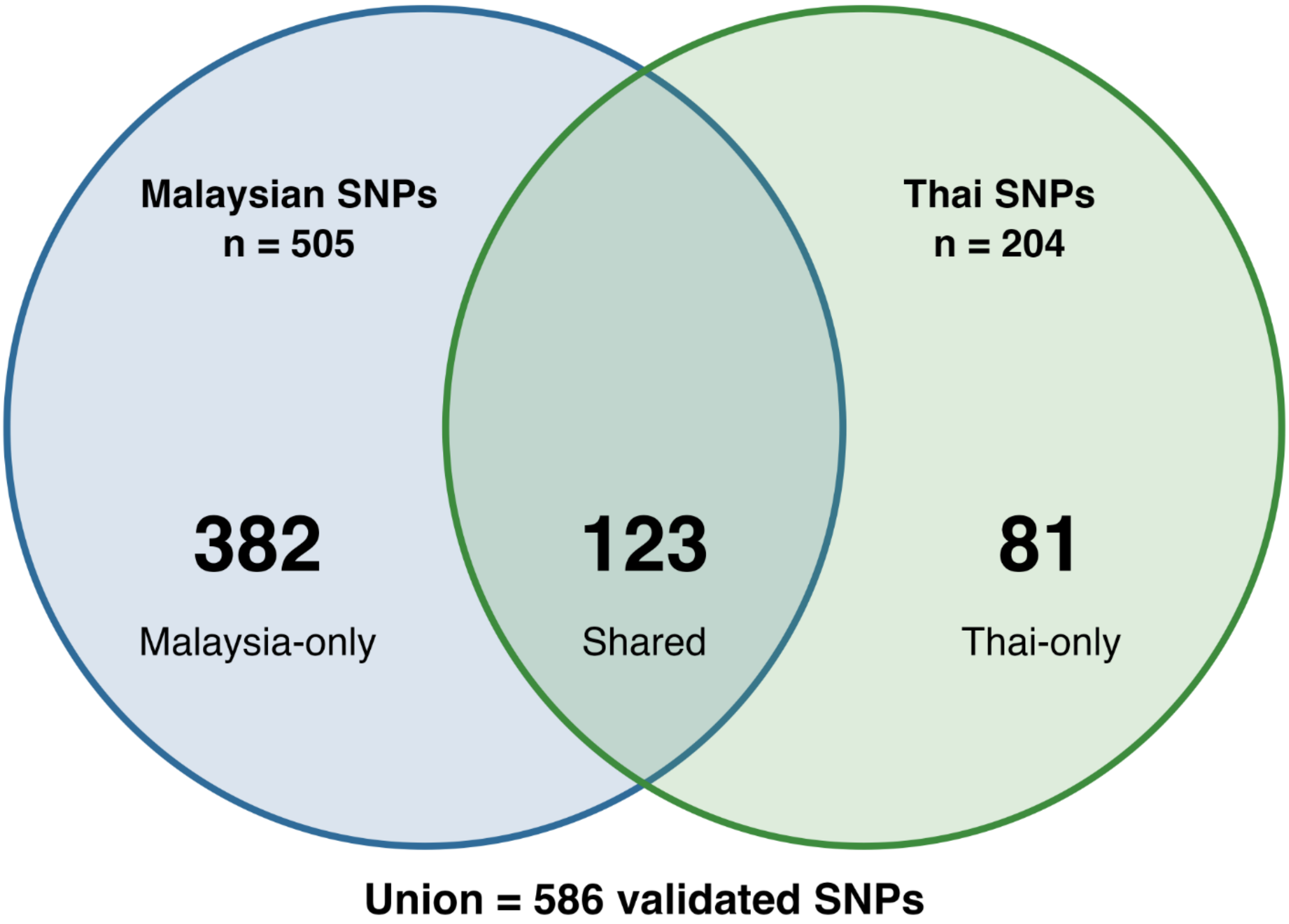

Supplementary Figure S5. Overlap between Malaysian and Thai variant positions across the 27 recovered amplicon markers. The published Malaysian WGS dataset contained 505 variable SNPs within the recovered amplicon markers, while 204 variant positions were identified among the Thai infections relative to the *P. knowlesi* H reference genome. Of these, 123 positions were shared, 382 occurred only among the published Malaysian WGS genomes, and 81 occurred only among Thai infections, yielding a combined 586 SNPs. The 204 Thai variant positions comprised 176 positions segregating among Thai infections and 28 positions fixed for a non-reference allele.

medRxiv preprint doi: https://doi.org/10.64898/2026.10.16.26365752; this version posted October 19, 2026. The copyright holder for this preprint (which was not certified by peer review) is the author/funder, who has granted medRxiv a license to display the preprint in perpetuity.
It is made available under a CC-BY 4.0 International license .

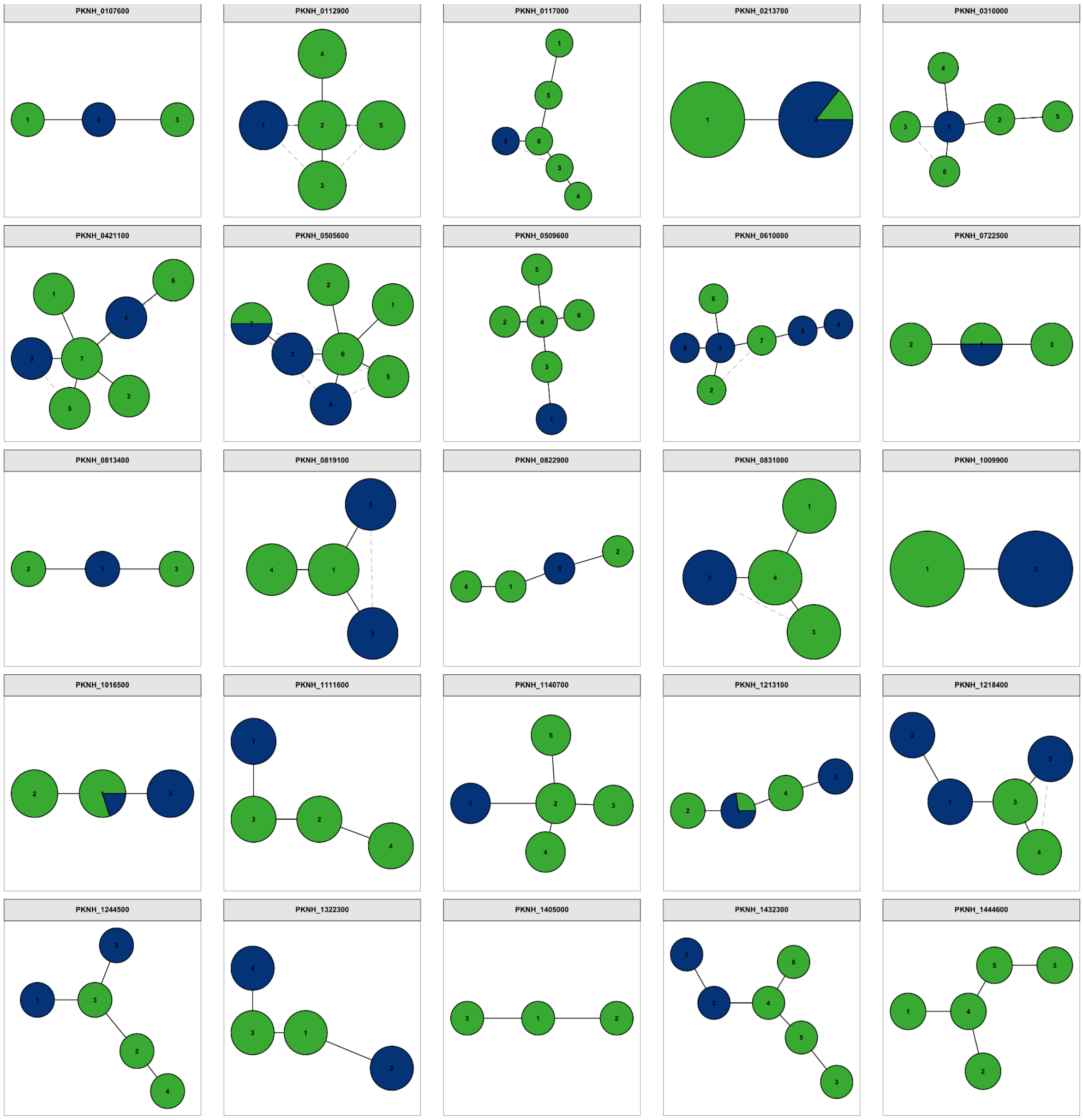

Supplementary Figure S6. Haplotype networks for the 25 polymorphic loci included in downstream analyses. Each panel represents one locus, and each node represents one haplotype observed across the 23 analyzed infections. Edges indicate mutational steps. Numbers within nodes correspond to haplotype identifiers as inferred by the nanopore-adapted HaplotypR pipeline.
